# Wearable Monitoring Devices for Assistive Technology: Case Studies in Post-Polio Syndrome

**DOI:** 10.3390/s140202012

**Published:** 2014-01-24

**Authors:** Giuseppe Andreoni, Marco Mazzola, Paolo Perego, Carlo Emilio Standoli, Simone Manzoni, Luca Piccini, Franco Molteni

**Affiliations:** 1 Design Department, Politecnico di Milano, via G. Durando 38/A, Milan 20158, Italy; E-Mails: marco.mazzola@polimi.it (M.M.); paolo.perego@polimi.it (P.P.); carloemilio.standoli@gmail.com (C.E.S.); simone.manzoni@mail.polimi.it (S.M.); 2 SXT-Sistemi per Telemedicina s.r.l., via M. D'Oggiono 18/A, Lecco 23900, Italy; E-Mail: lpiccini@sxt-telemed.it; 3 “Villa Beretta” Rehabilitation Center, Valduce Hospital, Via N.Sauro, 17 - 23845 Costa Masnaga (LC), Italy; E-Mail: fmolteni@valduce.it

**Keywords:** wearable monitoring, lower-limb orthosis, post-polio syndrome, impedance cardiograph, six-minute walking test

## Abstract

The correct choice and customization of an orthosis are crucial to obtain the best comfort and efficiency. This study explored the feasibility of a multivariate quantitative assessment of the functional efficiency of lower limb orthosis through a novel wearable system. Gait basographic parameters and energetic indexes were analysed during a Six-Minute Walking Test (6-MWT) through a cost-effective, non-invasive polygraph device, with a multichannel wireless transmission, that carried out electro-cardiograph (ECG); impedance-cardiograph (ICG); and lower-limb accelerations detection. Four subjects affected by Post-Polio Syndrome (PPS) were recruited. The wearable device and the semi-automatic post-processing software provided a novel set of objective data to assess the overall efficiency of the patient-orthosis system. Despite the small number of examined subjects, the results obtained with this new approach encourage the application of the method thus enlarging the dataset to validate this promising protocol and measuring system in supporting clinical decisions and out of a laboratory environment.

## 1. Introduction

Post-Polio Syndrome (PPS) is a neuromuscular disease that is characterized by muscular weakness and pain, abnormal weariness and muscular atrophy, likely due to the viral destruction of the medullar motor neurons and to the consequent chronic degeneration of the motor units endings. This condition afflicts PPS patients up to many years after the recovery from the first acute attack of the poliomyelitis virus and may eventually involve a highly disabling loss of motor functionality [1].

A physiological walking activity requires muscular strength, joint mobility and coordination of the central nervous system. PPS cause a severe deficit in one or more of these abilities, jeopardizing the normal motor pattern. Major symptoms of PPS are a severe weakening of the motor system and a remarkable trouble in respect to a physiological walking activity. The lower extremities, shoulders and low-back are the most common sites of dysfunction [2]. In particular, lower-limb muscular weakness emerges during a normal walking activity, wherein the step symmetry and rate results are altered.

Knee-Ankle-Foot Orthosis (KAFO) or Ankle-Foot (AFO) Orthosis are often recommended to support and improve motor efficiency in polio-survivors with lower limb disability [3]. However, a badly designed orthosis may be less attractive, may fail prematurely and may be detrimental for the patient. The ability of orthosis to lessen the patients' symptoms varies with the anatomical location of the impairment and with its severity [4]. For instance, KAFO users must adopt abnormal gait patterns to compensate for the knee motion constraints imposed by the brace. These abnormal compensatory patterns may lead to soft tissue injury and joint dysfunction at the hip and at the lower back, which may cause pain and reduction in the range of motion [5]. Walking with an immobilized knee also reduces walking efficiency by 24%, thereby leading to premature fatigue and limiting the distance a user can walk [6]. Surveys have shown that increased energy demand from using KAFO is one of the major reasons for which KAFO rejection rates range from 60% to nearly 100% [7]. A new type of KAFO, named Stance-Control Knee-Ankle-Foot Orthosis (SCKAFO), has been designed to allow free knee motion. It improves gait symmetry/kinematics, mobility, and requires less compensatory movements [5,8], and less energy expenditure when walking [9,10].

The functional assessment of the orthosis requires a careful multifactorial analysis based on subjective (patient self-report) and objective parameters. The effectiveness of patients' AFO/KAFO orthosis is commonly evaluated either by static joint-flexion of the orthosis or by visual observation of the brace on the patient during gait [11]. A number of commercially available goniometers and electro goniometers may be used to measure the range of motion of a joint. However, electromechanical systems used to quantify motion at various joints during gait are: (i) not portable enough, with hardware that may be obtrusive; (ii) not application specific, *i.e.*, a clinician has to modify his/her clinical protocol to make adequate use of the device; and, (iii) expensive (e.g., camera-based optical measurement systems) [12,13].

Wearable technologies (WT) have been exploited for gathering biological data in the long-term monitoring field [14]. They have been used to recognize motor activity and to observe patients during rehabilitation [15,16]. The use of WT in the study and management of patients affected by movement disabilities is very promising, because it may improve the objectivity of the analysis through quantitative measures of the pathological events. Furthermore, it may overcome the limits of the existing measurement systems, giving the opportunity to reach mid or long-term data recordings both in clinical and home environments with a non-invasive low-cost method.

Energy expenditure measurements, such as the physiological cost index (PCI) [17], the total heart beat index (THBI) [18] and the oxygen cost/rate have proven to be a reliable index for quantifying penalties imposed by gait disability [9]. Impedance cardiography (ICG) has been exploited in the last decade as a well established method to provide a non-invasive continuous measure of the stroke volume in resting or mild exercise conditions [19–21].

Therefore, a relatively inexpensive, portable unit which can be easily incorporated into an orthotic evaluation protocol based on gait analysis was proposed. The post-processing software was devised to perform an application-specific assessment of the orthosis through acceleration measurement. Inertial data were processed to obtain gait basographic parameters usually provided by an optoelectronic system, like step/stride duration, length and rate. Moreover, the proposed wearable system was designed to support mechanical measures with bio-energetic data based on the acceleration and cardiac output signals.

A reporting method able to optimize the information provided through different bio signals was combined with a versatile device/electrodes arrangement. The integration of the acquired data may provide a more complete and contextualized information about the orthosis efficiency without the redundant use of two or more measuring systems with non-specific post-processing applications and different acquisition protocols.

The present study aimed to assess the feasibility of a wearable unobtrusive approach to evaluate the kinematical/energetic efficiency of a lower limb orthosis through both basographic parameters and metabolic expenditures during a standard gait analysis protocol (6-MWT). The potentialities of this new method were gauged by investigating four clinical cases of subjects affected by the Post-Polio Syndrome and to quantitatively assess the performances of the orthosis proposed by the caregivers.

## 2. Experimental Section

### 2.1. Subjects

Four PPS subjects (two males and two females, age between 45 and 67 years) participated in the current study. All the subjects had been affected by poliomyelitis in their childhood and were showing Post-Polio Syndrome symptoms. The previously recorded values of Body Surface Area (BSA), Body Mass Index (BMI), Basal Energy Expenditure (BEE), Predicted Walking Distance (6MWD) and Lower Limit of Normal distance (LLN) for each subject are shown in Table 1. The previous use of a lower limb orthosis (AFO/KAFO) was also reported.

The study was approved by the competent Institutional Review Board and Ethical Committee. Subjects were properly informed about testing procedures, personal data treating and aims of the research, and they provided informed consent before participation.

### 2.2. Instrumentation

**Figure 1.**
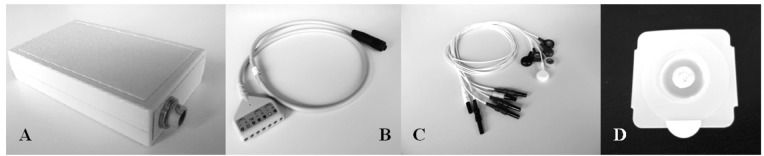
Wearable components of the ECG/ICG/acceleration cardiographic system. (**A**) Data-logger (117 × 70 × 23 mm); (**B**) Holter main cable. (**C**,**D**) Cable set and Cleartrace^TM^ adhesive electrodes (ConMed Corporation, Utica, NY, USA).

A prototype (Figure 1) derived from a commercial wearable polygraph (Phedra, SXT-Sistemi per Telemedicina, Lecco, Italy) was used to collect bio signals. This monitoring device was composed by an analogical circuit for the acquisition of ECG and ICG signal, and a digital board that provided data digitalization and transmission. The data-logger also contained a 3D acceleration sensor (MicroElectroMechanical System (MEMS), LIS3L06AL, STMicroelectronics, Geneva, Switz., range: ±6 g, sensitivity: 1.6 g/V) and was fixed on the patients' trunk through an elastic belt. The data-logger was powered by a rechargeable LiIon battery and provided 6-channels data to a remote processing unit through Bluetooth^®^ class II transmission module (PAN1540, Panasonic, Osaka, Japan).

The sampling frequency was set at 128 Hz. An on-line band-pass filtering (0.5–40 Hz) was performed on the ECG signal to reduce muscular, cable artefacts and high frequency noise. A double filtering (low-pass 1.7 Hz, band-pass 0.08–15 Hz) was applied to the ICG signal to find the base impedance *Z_0_* and the impedance variation *ΔZ*.

The wearable polygraph transmitted the signals via Bluetooth^®^ wireless connection to a PC running a dedicated on-line acquisition software (SXT-Sistemi per Telemedicina, Lecco, Italy) which provided data saving and real-time display of the signals features.

### 2.3. Acquisition Protocol

Each subject was tested through the six-minute-walking test (6MWT), which has been used as an evaluation tool for monitoring the functional motor behaviour of both healthy and pathological subjects [22,23]. The 6MWT measures the distance covered (6MWD) while walking along a straight direction over a six-minutes interval. The 6MWD is correlated to the disease severity and to the possible motor alterations induced by the orthosis.

Each individual was tested twice: at the beginning of the rehabilitative period (*t_0_*), before wearing the orthosis; and at the end of the rehabilitative training (*t_1_*), while using the orthosis. The subject was allowed to walk at a self-selected speed and to rest if he/she needed it. Each session was divided in order to monitor ICG/ECG signals under three different conditions:

(1) at rest in a sitting position (5 min), to measure the basal metabolic expenditure;
(2) under effort, during the 6MWT;
(3) after the exercise, in a sitting position (5 min).

**Figure 2.**
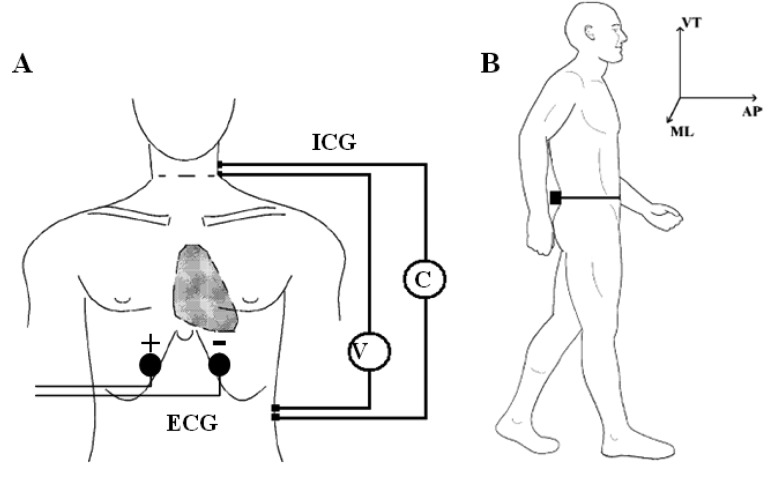
Experimental set-up. (**A**) Electrode positioning: C is the current injecting electrodes and V the voltage measuring electrodes for the impedance cardiograph while the ECG bipolar derivation is fixed under the sternum; (**B**) The wearable unit and the acceleration sensing axes (ML: medium-lateral, AP: antero-posterior, V: vertical).

Accelerometric monitoring was also carried out in condition (2). Before the acquisition, the subject was prepared by steadily fixing the wearable polygraph at the lumbar area (L2,L3) through an elastic belt. This was done to ensure a measurement of the thorax-pelvis accelerations that minimizes the noise component due to MEMS vibrations and bumps. The (L2,L3) area was also chosen because it roughly represents the nearest position to the centre of mass of the body [24]. A single bipolar derivation was used to record ECG signal. Two electrodes were positioned five centimetres under the sternum with a mutual distance of 15 cm. This simplified setting was intended to measure only the hearth rate (HR), without considering other ECG physio-pathological features (Figure 2A).

ICG measures the beat-to-beat changes of thoracic bioimpedance via four dual sensors applied on the neck and thorax in order to calculate stroke volume (*SV*). The experimental set-up consisted of four spot electrodes on the left side of the body (Figure 2B—lateral spot array) [21], a less intrusive configuration than the conventional 8-electrodes or band-electrodes ones [19,20]:

(1) injection electrodes: one on the upper side of the neck and one on the flank, about five centimetres under the xiphoid process;
(2) sensing electrodes: one fixed five centimetres under the injection electrode on the neck and one near the xiphoid process, above the injection electrode.

### 2.4. Data Processing

The wearable device was able to transmit the following bio signals to a remote processing unit:

(1) Antero-Posterior (AP) accelerations (*ACC_AP_*);
(2) Medio-Lateral (ML) accelerations (*ACC_ML_*);
(3) Vertical (V) accelerations (*ACC_V_*);
(4) Basic thoracic Impedance *Z_0_*;
(5) Impedance variation *ΔZ*;
(6) ECG potential.

The aim of the post processing algorithm was to provide a selection of indexes concerning gait time-space parameters and energy expenditure from acceleration and ICG/ECG signals respectively. A comparative analysis was carried out in order to provide quantitative information about the functional efficiency of the worn orthosis.

#### 2.4.1. ECG Signal Processing

A batch QRS detector based on the one proposed by Pan *et al.* [25,26] was used to extract the heart rate (HR). This solution allowed to detect the QRS complex and to identify the R-peak occurrence.

#### 2.4.2. ICG Signal Processing

Two Butterworth low-pass filters (third order) with cut-off frequencies of 8 and 2 Hz were used to pre-process *ΔZ* and *Z_0_* signals, respectively.

**Figure 3.**
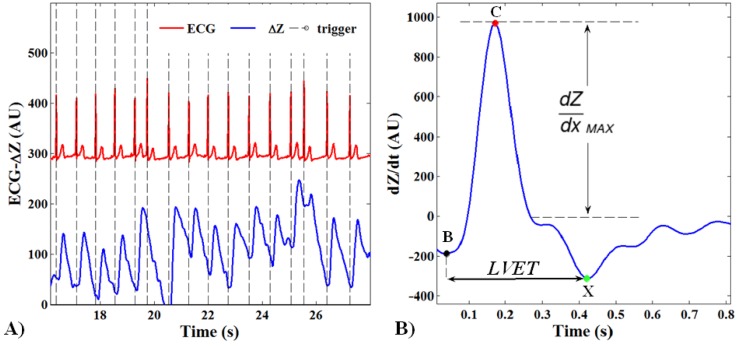
Example of the ICG processing workflow. (**A**) The *ΔZ* signal is shown with its respiratory component, the identified R peaks of the ECG signal trigger the subsampling on *ΔZ*; (**B**) the interesting points B-C-X automatically recognized on the averaged *ΔZ* are shown.

Impedance changes related to heart cycles were separated from the superimposed noise caused by respiration through a synchronous averaging method [27]. The whole ICG signal *ΔZ(t)* was divided in N subsamples of changeable length depending on the temporal triggers provided by the R-peaks of the ECG signal (Figure 3A). The out coming N subsamples were summed and averaged.

The stroke volume (*SV*) was estimated through the Bernstein-Lemmens equation (Equations (1) and (2)) after the automatic recognition of the interesting points on the dZ/dt averaged waveform (Figure 3B):

(1) B: aortic valve opening;
(2) C: systolic maximal (dZ/dt_MAX_);
(3) X: aortic valve closing.

(1) $${SV}_{n}=V_{c}*\sqrt{\frac{{\left(dZ(t)/dt\right)}_{\mathrm{MAX}}}{Z_{0}}}*\text{LVET}$$

where *V_C_* is the conduction volume defined as the ratio between the intrathoracic blood volume *V_ITEV_* (ml) and the square trans-thoracic conduction index *ζ* (adimensional). The left ventricle ejection time (*LVET*) was given by the B-X time distance:

(2) $$V_{C}=\frac{V_{\text{ITEV}}}{\zeta^{2}}=\frac{16*W^{1.02}}{\zeta^{2}}$$

For *Z_0_* < 20 Ω, 0 < *ζ* < 1 whereas for *Z_0_* ≥ 20 Ω, *ζ* = 1. *W* is the body mass of the subject [28].

#### 2.4.3. Acceleration Signal Processing

Having one single accelerometer we did not calculate kinematic parameters of lower limbs during gait or apply algorithms for trajectory computation as in other multisensory rehabilitation approaches [29,30]. The acceleration signal processing aimed to assess basographic parameters for gait evaluation. The AP, ML, and V components were low-pass filtered with a cut-off frequency of 3 Hz (Butterworth, third order). The algorithm made use of the geometrical solution proposed by Moe-Nilssen *et al.*, [31] to correct the possible changes in the directions of the sensing axes of the accelerometer.

**Figure 4.**
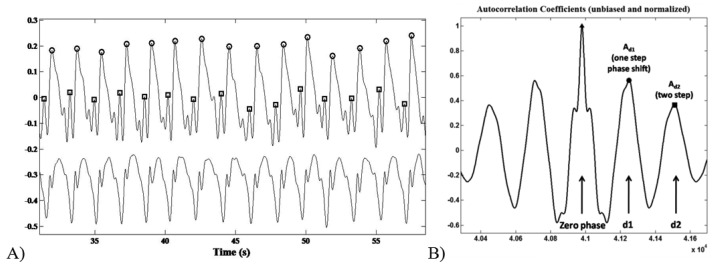
(**A**) Left-Right contact detection process. Round and square markers identified the left and right foot-ground contacts respectively, within the AP acceleration signal (upper line) in g. Peaks were discriminated on the basis of the local pattern of the ML signal (lower line) in arbitrary unit for data visualization; (**B**) Middle section of the unbiased and normalized autocorrelation sequence of vertical trunk acceleration during normal walking: d1 and d2 represent a phase shift of the first and the second step (of the contralateral limb), respectively.

The recognition of the foot-ground contact was based on the acceleration peaks identification within the AP channel. The ML signal trend was useful to discriminate between left and right stance. Step/stride duration (s), cadence (step/min), mean velocity (m/min) and step/stride length (m) were determined starting from the estimation of the period between an acceleration peak and the following one (Figure 4A).

The acceleration signal on the vertical (V) and Medium-Lateral (ML) axes during the six-minute-walking test reflected a typical periodic pattern. The middle section of an unbiased and normalized autocorrelation sequence of vertical trunk acceleration during normal walking was analysed. Within a gait cycle the first and the second autocorrelation peaks (after the zero-phase peak) reflect the relation between right and left steps of the contralateral limbs, respectively.

As previously reported by Moe-Nilssenet *et al.*, [32], since the first dominant period represents a phase shift of one step (*d1*), the autocorrelation coefficient at the first dominant period *A_d1_* is an expression of the regularity of the acceleration signal between neighbouring steps (Figure 4B). Hence, we performed a regularity/symmetry analysis on the acquired step acceleration pattern, in which for the vertical axis closeness of each of *A_d1_* and *A_d2_* to 1.0 reflects step and stride regularity, respectively, while closeness of *A_d1_*/*A_d2_* to 1.0 reflects symmetry. On the contrary, for the ML axis, *A_d1_* ∼ −1 and *A_d2_* ∼ 1 represents the step/stride regularity, whereas *A_d1_*/*A_d2_* ∼ −1 shows the step symmetry.

During the walking test each subject covered the same path for several times: the parameters inferred through signals acquired along the same path were averaged. The acceleration signal during rest breaks were discarded.

#### 2.4.4. Energy Expenditure Indexes

In this study we wanted to provide a comprehensive vision of the human-orthosis system both from biomechanical and metabolic (energetic) point of view. For this reason we tried to find a specific index concerning this second aspect, but in literature we did not succeed in finding a solution directly applicable in our case study. Thus our methodological choice, in accordance to the clinicians' suggestion, was to consider the different indexes reported in literature to assess motor capabilities and compatible (for their computation with the adopted experimental setup. Metabolic indexes were derived by the unobtrusive measures provided by the polygraph, without the use of direct/indirect calorimetric measurements or respiratory gas analysis. In particular, the following indexes were estimated:

(1) Energy Expenditure due to physical activity (*EE_act_*);
(2) Physiological Cost Index (*PCI*);
(3) Total Hearth Beat Index (*THBI*);
(4) Oxygen rate (*O_2_ rate*) and oxygen cost (*O_2_ cost*).

*EE_act_* could be inferred through the acceleration data, since significant correlations between energy expenditure and accelerometer readings were found under controlled conditions. Data from studies on gait analysis and ergonomics have demonstrated a linear relationship between the integral of the absolute value of body acceleration and energy expenditure [33,34]. Bouten *et al.* [35] obtained a linear relation between *EE_act_* (W/kg) and the acceleration modulus integral in the FB direction (*a_x_*). In their experimental setup based on a triaxial accelerometer fixed on a belt and worn in the low-back area, the most accurate estimation of *EE_act_* was achieved during walking by integrating the absolute value of unidirectional acceleration in antero-posterior direction (IAA_X_):

(3) $$EE_{\text{act}}=-0.176+0.085*IAA_{x}$$

where *IAA_x_* was defined as:

(4) $$IAA_{x}=\int_{t=0}^{T}\left|a_{x}\right|dt$$

Studies on gait analysis and ergonomics have demonstrated a linear relationship between the integral of the absolute value of body acceleration in the antero-posterior direction and energy expenditure [33–35].

*PCI* is a clinical tool for the evaluation of the energetic consumption based on the linear relation between the oxygen consumption and the heart rate in sub-maximal load conditions. The *PCI* (beats/m) index was calculated, according to MacGregor *et al.* [17], as:

(5) $$\text{PCI}=\frac{\text{WHR}-\text{RHR}}{WR}$$

*WHR* (beats/min) was the walking heart rate, *RHR* (beats/min) was the resting heart rate and *WS* (m/min) represented the walking speed. Two requirements were needed to this index to be correctly assessed: an *HR* steady-state condition must be reached during walk and rest stages and subjects must walk at their own preferred velocity [36]. A low *PCI* value may suggest an efficient gait from an energetic point of view.

Together with the *PCI* index, we used the total heart beat index (*THBI*) as a high repeatability energetic test under steady-state and no steady-state conditions [18]. It was calculated by dividing the total heartbeats during activity by distance travelled in meters.

The O_2_ indexes were inferred through the gait velocity measures on the basis of the regression equations proposed by Waters *et al.* [37], for a healthy adult subject:

(6) $$O_{2}\;\text{rate}=0.129*\text{V}+2.60$$

(7) $$O_{2}\;\text{cost}=0.129+\frac{2.60}{V}$$

where the rate of oxygen consumption is in mL/kg per min of *O_2_* and *V* equals the walking speed in units of meters per minute. The *O_2_ cost* per meter is directly related to the extent of the patient's gait disability. The *O_2_ rate* indicates the physiological effort of walking at the selected speed. Orthosis that substitute for lost muscle function or reduce lower extremity joint deformities can improve walking efficiency and the associated energy cost [9].

The algorithms for off-line signal processing and the evaluation of the kinematic/energetic indexes were implemented in Matlab^®^ (Mathworks Inc., Natick, MA, USA). These indexes were used to overcome experimental problems with ICG recordings. In fact the ICG signal is low and its quality is severely affected by wire motion and the presence of other bioelectrical signals. During the tests only in one patient's ICG quality was considered reliable for further data processing.

## 3. Results and Discussion

The whole of the resulting parameters was summarized in a comparative table in which were shown both kinematic and energy expenditure indexes for each subject.

### 3.1. Kinematic Results

Acceleration patterns showed the gait performance parameters for a 6MWT both with and without a KAFO orthosis and after a training period (Table 2). Subjects 1,3,4 manifested lower gait performances wearing a KAFO orthosis, including a reduction in covered distance, mean velocity and cadence. Subject 2 was the only one that improved her velocity and cadence by 15% and 10%, respectively; step durations were lower and the right-left difference showed a 12% decrease.

**Figure 5.**
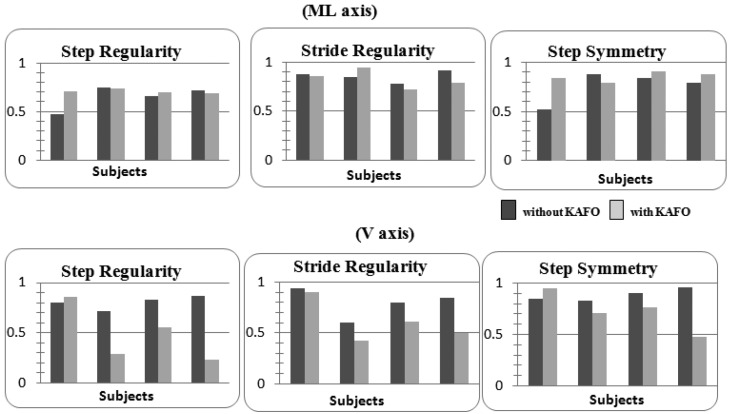
Comparison of step/stride regularity and symmetry evaluated both for the ML and V acceleration sensing axes. Values were normalized and expressed as non-dimensional indexes ranging from 0 to 1. Light grey and dark grey were used to discriminate between the trials carried out with and without the orthosis, respectively.

The histograms with the inferred values for step/stride regularity and symmetry were reported both for the ML and V axes (Figure 5). In the ML axis we focused our attention on the recovery of a symmetrical gait that is important for reducing both impact forces on the Post-Polio limb and postural corrections during gait. For the ML axis, subject 1 showed an increased step regularity (+34%) whereas the other subjects manifested lower performance variations (less than 10%, so we can consider it as constant in the other patients). Step symmetry is significantly increased (+38% in subject 1, about 10% in subjects 3 and 4) while wearing the orthosis in three subjects, while patient 2 presents a 10% reduction. This result was also backed by the step symmetry/regularity evaluation on the V axis, in which subject 1 showed the same improvement (although with a lower percentage) with respect to the other subjects that highly decreased step/stride regularity (between 30% and 73%) and symmetry (between 12% and 48%). This reduction of step regularity was probably due to the new orthosis they were asked to wear; so they needed to get acquainted to the new situation and should be recovered during training.

### 3.2. Cardio-Vascular Results

The HR and the CO evaluated through the ECG/ICG signals were reported for basal, effort and recovery conditions (Table 3). The difference between the mean HR under basal and effort conditions increased from 8.6% to 28% when the subjects wore the orthosis. Only subject 1 showed a decrease by 50% of the same quantity. The presence of the orthosis caused the differences between the CO in recovery and basal condition to be greater on all subjects (at least 16%). The CO could not be assessed but in one case, under effort condition due to the high noise level on the cardiographic measurements during the walking test.

### 3.3. Energetic Results

**Figure 6.**
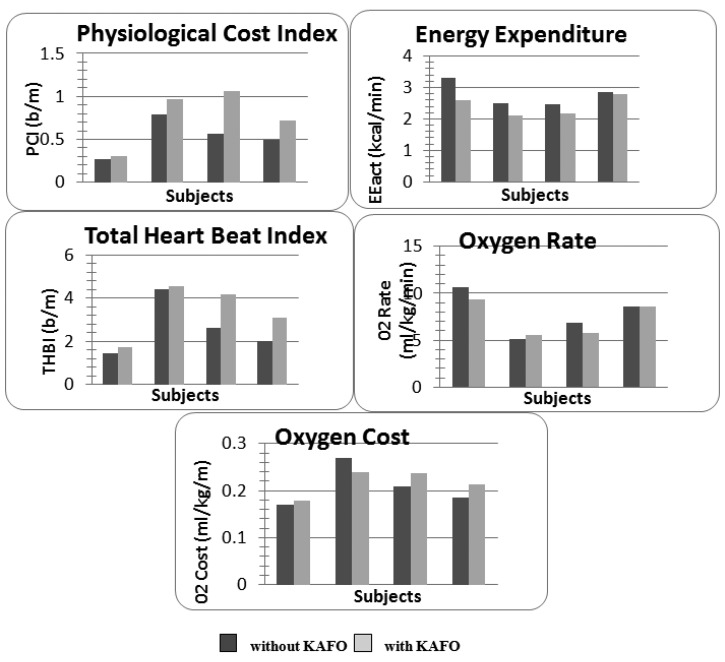
Comparison of the energy expenditure indexes evaluated through the ICG/ECG and acceleration signal. Light grey and dark grey were used to discriminate between the trials carried out with and without the orthosis, respectively.

The indexes referring to the energy expenditures and to the cardiac/respiratory activity during the walking test were reported in Figure 6. The energy consumption evaluated on an accelerometric basis (*EE_act_*) decreased by (mean ± SD on the whole sample) 12.8 ± 8.2% when the subject wore a KAFO orthosis. On the contrary, the physiological cost index and the total heart beat index showed a global loss of performances for each subject (PCI: 20.9 ± 18%, THBI: 14.8 ± 15.8%) when an orthosis was used. No main effect was found regarding the oxygen consumption: subjects 1,3,4 improved the *O_2_ rate* (9.3 ± 6.7%), though they showed an *O_2_ cost* enhancement (7.5 ± 6%).

From the results we can note a contradiction in the parameters according to the stated datum: if we consider physiology (*i.e.*, ECG and HR signal), the use of the KAFO orthosis produced an increase of the metabolic effort as evidenced by the increase in PCI and THBI. In fact subjects 2 and 3 covered the same distance with and without the orthosis during 6MWT but HR was higher both resting and walking. This situation was even worse for subjects 1 and 4 who also covered a shorter distance with higher HR. Instead the indexes computed starting from accelerations evidenced an increased energetic performance (*i.e.*, reduction of metabolic energy for the same activity). For a better understanding of the indexes reliability a 3-months and 6-months control should be carried out in order to verify the training with the KAFO and the coherency of the indexes. According to these preliminary findings, we decided to adopt the *EE_act_* index for assessment on the early adoption of the orthosis. In fact we consider the biomechanical immediate improvement as more significant with respect to the metabolic one with is related to training and exercise, thus to be evaluated in a regime condition.

A good quality of life cannot be separated from the total or partial recovery of the functional ability. In the particular case of the post-polio subjects, the use of an orthosis applied to the weakened lower limb may lead to a significant improvement of the cardiovascular energetic and mechanical parameters. This is necessary to recover gait ability and to reduce muscular and joint pain, allowing a satisfying level of autonomy. The deficit of an objective and reliable assessment of the biomechanical solution involved may be covered by a small cost-effective wearable device. It was able to provide a large spectrum of objective data, helping to assess the functional performances of an orthotic tool and supporting the clinical evaluation with an intuitive report board.

The whole of the kinematical/energetic parameters were evaluated through ICG, ECG and acceleration measures provided by the wearable system, allowing the clinical staff to suggest an optimal rehabilitative solution based on the objective functional assessment of the KAFO orthosis. The inferred indexes were previously defined and assessed for their reliability. In our opinion, the overall inaccuracy from intrinsically relying on indirect methodologies may be mitigated by the use of a comparative analysis that may supply each single uncertainty with a redundant and contextual information. At this stage, we focused on a preliminary approach to a multivariable methodology that made use of several nonspecific indexes. A helpful improvement should consider a case-dependent set of parameters which could be strictly related to the pathology characteristics and to the rehabilitative tool (*i.e.*, AFO, KAFO, custom-made or not, *etc.*).

An optimized approach requires overcoming a number of limiting factors due to the combined use of the wearable device and of the proposed method. One of the major problems was represented by movement artefacts, whose spectra are unknown and may sometimes overlap the impedance signal spectrum. Barrios *et al.* [23] recently claimed that this problem may be solved by an adaptive filtering technique. The evaluation of the cardiac output during the walking stage was strongly jeopardized by the movement artifact noise. The post-effort monitoring suffered of the same inconvenient, but with a higher signal to noise ratio. Therefore it was necessary to manually select the *ΔZ* signal for several trials to discard the highest noise features, averaging the enhanced ones.

Efforts should be spent to reduce noisy patterns due to electrodes positioning and fixing. The use of cables may be avoided through a sensorized shirt, with the data-logger fixed in a pocket at the lumbar level and electrodes inside the shirt fabric. This solution requires optimizing the electrode technology to be “active” for the CO measurement.

The subject sample number should be increased in order to obtain both a stronger reliability validation and of the described method and a reference dataset for different orthoses to draw general guidelines. Two additional issues must be considered analysing the results of the trials: the short training period with the KAFO (at least 2 days) in respect to a consolidated gait behaviour strengthened over time and the use of a non-custom-made orthosis. Greater values of velocity and cadence obtained without the orthosis may not necessarily mean that the subject showed a better gait pattern, but could be explained in terms of patient habits of walking in a certain manner. The repetition of the trials after 3-weeks training time should be worth considering to complete the protocol.

## 4. Conclusions/Outlook

The quantitative measures and the inferred indexes provided through the wearable polygraph allowed an efficiency assessment of the orthosis from an energetic and kinematic point of view. The device required a minimal invasiveness, confined to the use of the adhesive electrodes applied on the skin. The semi-automatic post-processing algorithm reduced the assessment time and was able to summarize and compare the results of energy and gait time-space studies performed in patients with specific neurologic and orthopaedic disabilities.

Thanks to a tri-axes accelerometer, it could be possible to study the walking activity of pathological subjects. The system provided less information in respect to the standard gait analysis, but they were referred to a continuous locomotion and were closer to everyday life conditions than a laboratory environment. In order to assess the reliability of the wearable system in respect to the gold standard and to build up a standardized measurement tool, the trial database will be eventually increased.

## Figures and Tables

**Table 1.** Individual characteristics of the analysed subjects.

**Subject**	**Age**	**Gender**	**BSA (m^2^)**	**BMI (kg/m^2^)**	**BEE (kcal/min)**	6MWD* (m)	**LLN (m)**	**Orthosis**
1	54	M	2.00	30.5	1.20	540	387	-
2	45	F	1.76	27.7	0.98	495	356	AFO
3	67	M	2.00	28.4	1.13	509	356	Stick
4	57	F	1.73	27.2	0.92	477	338	SCKAFO

* The predicted distance for an healthy adult subject can be inferred by its individual characteristics [22].

**Table 2.** Kinematic indexes measured during the monitoring trials through the 6MWT and the acceleration analysis (R = right, L = left).

**Subject**		**Covered Distance (m)**	**Mean Velocity (m/min)**	**Cadence (step/min)**		**Stride Length (m)**	**Step Length (m)**		**Stride Duration (s)**	**Step Duration (s)**		**FB Foot-Contact Peak (g)**	
	
							R	L		R	L	R	L
1	no KAFO	365	63	97	1.30		0.71	0.59	1.24	0.68	0.56	0.37	0.35
	KAFO	305	53	89	1.20		0.65	0.55	1.34	0.73	0.61	0.33	0.28

2	no KAFO	117	20	58	0.68		0.49	0.19	1.49	1.49	0.60	0.14	0.34
	KAFO	116	23	64	0.73		0.51	0.21	1.36	1.36	0.57	0.16	0.33

3	no KAFO	100	33	67	0.99		0.42	0.57	1.80	0.76	0.68	0.26	0.17
	KAFO	100	24	61	0.81		0.28	0.53	1.99	1.04	1.30	0.33	0.24

4	no KAFO	280	50.1	94	0.98		0.41	0.57	1.27	0.53	0.74	_^b^	_ ^b^
	c-KAFO^a^	184	31.8	79	0.77		0.36	0.40	1.52	0.72	0.80	_ ^b^	_ ^b^

a Custom-made orthosis;

b n.d.

**Table 3.** Mean values of the ECG/ICG parameters measured under different trial conditions.

**Subjects**		**Mean Heart Rate (b/min)**			**Cardiac Output (l/min)**		

		**basal**	**effort**	**recovery**	**basal**	**effort**	**recovery**
1	no KAFO	74	90	77	5.86	_	6.20
	KAFO	81	89	81	5.61	_	6.31

2	no KAFO	69	85	67	3.82	_	4.03
	KAFO	81	103	82	4.48	6.15	4.86

3	no KAFO	68	86	71	5.14	_	6.12
	KAFO	74	99	78	5.91	_	6.73

4	no KAFO	71	94	73	5.10	_	7.21
	c-KAFO*	77	98	79	5.21	_	7.76

* Custom-made orthosis.
